# Highly Efficient Near-Infrared Detector Based on Optically Resonant Dielectric Nanodisks

**DOI:** 10.3390/nano11020428

**Published:** 2021-02-08

**Authors:** Reza Masoudian Saadabad, Christian Pauly, Norbert Herschbach, Dragomir N. Neshev, Haroldo T. Hattori, Andrey E. Miroshnichenko

**Affiliations:** 1 School of Engineering and Information Technology, University of New South Wales, Canberra, ACT 2600, Australia; r.masoudiansaadabad@student.adfa.edu.au (R.M.S.); h.hattori@adfa.edu.au (H.T.H.); 2 IEE S.A., Bissen L-7795, Luxembourg; christian.pauly@iee.lu (C.P.); norbert.herschbach@iee.lu (N.H.); 3 ARC Centre of Excellence for Transformative Meta-Optical Systems (TMOS), Electronic Materials Engineering, Research School of Physics, The Australian National University, Canberra, ACT 2601, Australia; dragomir.neshev@anu.edu.au

**Keywords:** NIR photodetector, bandwidth, dielectric nanodisks

## Abstract

Fast detection of near-infrared (NIR) photons with high responsivity remains a challenge for photodetectors. Germanium (Ge) photodetectors are widely used for near-infrared wavelengths but suffer from a trade-off between the speed of photodetection and quantum efficiency (or responsivity). To realize a high-speed detector with high quantum efficiency, a small-sized photodetector efficiently absorbing light is required. In this paper, we suggest a realization of a dielectric metasurface made of an array of subwavelength germanium PIN photodetectors. Due to the subwavelength size of each pixel, a high-speed photodetector with a bandwidth of 65 GHz has been achieved. At the same time, high quantum efficiency for near-infrared illumination can be obtained by the engineering of optical resonant modes to localize optical energy inside the intrinsic Ge disks. Furthermore, small junction capacitance and the possibility of zero/low bias operation have been shown. Our results show that all-dielectric metasurfaces can improve the performance of photodetectors.

## 1. Introduction

Detection of infrared (IR) photons is an essential component of multifunctional optoelectronic technologies with versatile applications in different areas, from biosensing [1] and imaging [2] to optical communications [3] and computing [4]. Among the different types of detectors that exist on the market, more compact and sensitive optical sensors are required. For the detection of near-infrared (NIR) photons in optical network systems, germanium (Ge) is the best semiconductor due to its narrow bandgap, 0.66 eV at 300 K (i.e., 1.88 μm) [5]. Semiconductor photodetectors have several merits, such as compatibility with existing large-scale foundry technology and the capability of integration with signal processing in photonic integrated circuits.

One of the most common types of photodetectors is PIN diodes due to their compact size and large active area. An important parameter of photodetectors is bandwidth, which refers to the speed with which the detector responds to an incident of light and is a crucial parameter for the realization of high-speed optoelectronic devices. Miniaturizing the size of photodetectors is a way to decrease the internal capacitance, and thus to increase bandwidth, but on the other hand, it decreases light absorption (or quantum efficiency). Therefore, there is a tradeoff between quantum efficiency and bandwidth. A metasurface made of intrinsic semiconductor nanoparticles standing between p- and n-doped regions can optimize both the bandwidth and quantum efficiency of the photodetector. Furthermore, dielectric metasurfaces made of semiconductor nanoresonators promise a range of new phenomena and applications in the field of optoelectronics via the interference of electric and magnetic multipoles [6,7]. The confluence of nanophotonics and optoelectronics offers an unprecedented ability to control light–matter interactions. Dielectric metasurfaces allow the improvement of both the light collection efficiency [8] and bandwidth [9], which are two crucial components in the photodetector design. Many semiconductors have a high refractive index in the NIR and visible range of the electromagnetic spectrum that leads to unique optical properties such as a non-radiating anapole state localizing the electric field inside semiconductor nanoparticles [10]. Furthermore, unlike their plasmonic counterparts with ohmic losses, semiconductor nanostructures have a higher conversion efficiency of photons to charge carriers [11,12,13]. Such unique properties in conjunction with their small sizes can lead to the design and fabrication of high speed, multifunctional, and cost-effective photodetectors.

Here we propose a metasurface-based photodetector consisting of a subwavelength array of Ge PIN diodes. Germanium is compatible with the complementary-metal-oxide-semiconductor (CMOS) process and therefore, is a proper building material for photonic integrated circuits (PICs) high-volume foundry process. The performance of the PIN diode is studied via optical and electrical simulation at C-band (i.e., wavelengths 1530–1565 nm in optical communication systems). We first analyzed the optical properties of the metasurface, followed by the simulation of the steady-state and transient behaviors of a single unit of the device to obtain an electrical response. We showed that the photodetector exhibits a high bandwidth of 65 GHz at the reverse bias of 0.6 V. It also shows zero-bias operation, small junction capacitance, and high shunt resistance.

## 2. Device Configuration

**Figure 1:**
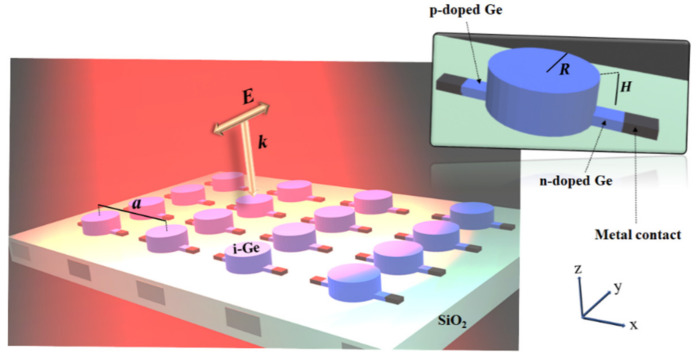
An array of PIN structures under the surface illumination of a y-polarized plane wave. Each pixel (see inset on the right) consists of a germanium (Ge) nanodisk of a radius R and a height H, sandwiched between two n- and p-doped Ge layers while metal contact circuits are embedded in the silicon dioxide layer.

We considered a lateral Ge PIN heterostructure for the realization of high-speed IR photodetection. Each pixel of the PIN diode consists of a Ge nanodisk (lightly n-doped by $10^{13}\;{\mathrm{cm}}^{-3}$), sandwiched between two n- and p-doped Ge layers with a moderate doping concentration of $10^{16}\;{\mathrm{cm}}^{-3}$. It has been shown that the doping concentration can affect the performance of photodetectors [14]. If the doping concentration is very high there will be increasing losses in absorption due to free carrier absorption and implantation-induced defect densities. However, with a doping concentration of $10^{16}{\;\mathrm{cm}}^{-3}$, the device has a large built-in field in order to extract the photogenerated charges, but the losses are negligible. The metallic contact circuits are embedded in a low-index substrate layer (silicon dioxide SiO_2_). The schematic of the structure and the different parameters of the device are shown in Figure 1. The device’s performance under surface illumination by plane wave with a polarization perpendicular to the contacts was studied. The radius and height of the intrinsic Ge cylinder (i-Ge) are, respectively, *R* and *H*, while the sizes of n- and p-doped regions are considered to be much smaller than that of the intrinsic layer, to guarantee that it does not affect the optical properties of the device (here we considered them as cubes of length 100 nm and height 50 nm). As shown in Figure 1, the parameter *a* is the lattice constant of the structure. Similar structures were recently fabricated, including an array of vertical Ge-Si heterojunction nanowires demonstrating narrowband photodetection [15,16] and nanopillar LEDs in the form of PIN junction [17].

## 3. Device Performance

Although surface illuminated photodetectors are widely used because of their low processing complexity, there is a tradeoff between their quantum efficiency and bandwidth. The speed of a photodetector is expressed by 3 dB bandwidth $f_{3db}$, the frequency where the output power decreases to half its maximum. The fraction of incident photons absorbed by a bulk Ge sample of thickness L is $1-e^{-\alpha L}$. The 3 dB bandwidth $f_{3db}$ can be expressed by $f_{3dB}≅0.382\;v_{s}/L$, where, for the carrier velocity, we used the saturation velocity $v_{s}\;=\;0.6\times 10^{5}\;m/s$, assuming that the electric field composed by the built-in field and the additional drift field generated by the non-zero bias voltage of the photodiode is sufficiently large. From this expression for $f_{3db}$ it can be understood that the bandwidth increases when the size L is decreased. This, however, also reduces the absorbed fraction of photons. For instance, a Ge device with $f_{3db}\;=\;100\;\mathrm{GHz}$ at wavelength 1.55 μm requires an intrinsic layer smaller than 250 nm. As $\alpha \;=\;459{\;\mathrm{cm}}^{-1}$ [18], such a thickness of Ge leads to a quantum efficiency of only 1%. To overcome these tradeoffs the thickness of the active Ge layer is typically in the order of nanometers to decrease the transit length of charge carriers and thus reduce their transit time. Still, the length of the active germanium layer is increased to micrometers to enhance quantum efficiency. For instance, recently a Ge waveguide photodetector of length 10 μm was proposed and reported $f_{3dB\;}\sim \;120\;\mathrm{GHz}$ [19]. However, thermal annealing used to optimize charge carriers’ velocity leads to a high dark current density of 80 $A/{\mathrm{cm}}^{2}$. Recently, a Ge waveguide PIN photodetector was reported that shows a cut-off frequency $f_{3dB\;}\sim \;67\;\mathrm{GHz}$ under bias -1 V [20]. The study reported a dark current of 4 nA and responsivity of 0.74 A/W for a Ge layer of size 14 μm. Another study reported a PIN photodiode based on lateral Si/Ge/Si heterojunction with a 3 dB bandwidth over 50 GHz [21]. A normal incidence Ge photodetector was recently reported that has a 0.39 A/W [22]. It has cut-off frequencies of 1 and 32 GHz for the designs with the mesa diameters of 60 and 10 μm, respectively. More recently, a plasmonic photodetector (3 μm in length) with a high 3 dB bandwidth $f_{3db\;}\sim 100\;\mathrm{GHz}$ and an internal quantum efficiency of 36% for the wavelength range 1270–1330 nm was reported, however, the device suffers from a high dark current of 0.1 μA [23]. Plasmonic materials (mainly gold and silver) also generate heat that can increase the temperature of plasmonic elements and eventually damage the device [24,25,26]. In Table 1, a breakdown of figures-of-merits for the performance characteristics of some Ge photodetectors is presented.

One of the solutions for the design of an optically thin photodetector with high light collection efficiency is to localize the optical energy inside the semiconductor and obtain maximum absorption. The maximization of optical power trapped inside nanostructures is possible by the efficient coupling of the light wave to high index semiconductor nanoparticles through the engineering of their optical resonant modes, as was suggested for all-dielectric Huygens’ metasurfaces [27], GaAs metasurfaces [28], and in anapole InGaAs nanolasers [29]. This efficient coupling is due to a resonant magnetic dipole response that has a comparable strength with the resonant electric dipole response. It arises from the fact that semiconductors are a low-loss material and, therefore, the electric field does not vanish inside particles, leading to a strong current flow. In this work, resonant modes engineering was used to realize photodetectors for the NIR spectral range, using Ge nanodisks.

**Table 1:** Performance of various Ge-based photodetectors. WG and NI refer to the waveguide and normal incidence photodetectors, respectively.

Structure	Highest Responsivity (A/W)	Dark Current	Highest 3 dB Bandwidth	Year	Ref
NI PIN	0.23	10 μA @ −1 V	-	2010	[30]
WG PIN	0.8	4 μA @ −1 V	120 GHz @ −2 V	2012	[19]
WG PIN	0.74	4 nA @ −1 V	67 GHz @ −1 V	2016	[20]
WG PIN	1.16 (for length of 40 μm)	10 nA @ −1 V	50 GHz @ −1 V	2017	[21]
NI PIN	0.39	$47\;\mathrm{mA}/{\mathrm{cm}}^{2}$ @ −1 V	32 GHz @ −1 V	2017	[22]
Plasmonic	-	0.1 μA @ −1 V	100 GHz	2018	[23]

### 3.1. Optical Design

**Figure 2:**
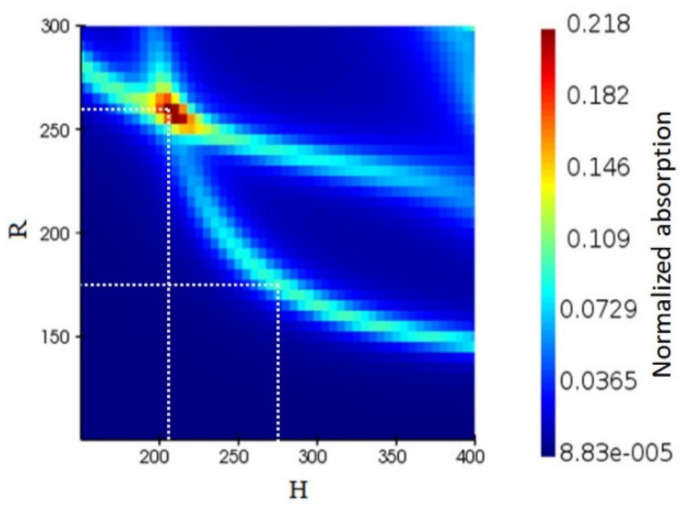
The normalized light absorption of the Ge PIN photodiode as a function of radius (R) and height (H) of the disk for the excitation wavelength of 1550 nm. The first is for *R_1_* = 175 nm and *H_1_* = 275 nm which corresponds to the magnetic dipole resonance. The second configuration is for *R_2_* = 260 nm and *H_2_* = 205 nm and corresponds to the overlap between the electric anapole state and magnetic dipole, giving rise to the absorption enhancement.

To calculate the optical response of the metasurface, finite-difference time-domain (FDTD) simulations were performed using Lumerical’s FDTD Simulation Solver. Initially, the optimized dimensions of the structure that gave the maximum absorption at the wavelength 1550 nm were obtained by a series of simulations for various values of the disk height, *H* and radius, *R*. The light absorption as a function of *H* and *R* is shown in Figure 2 where two electric dipole and magnetic dipole modes are observed. Two spots with high absorption are marked by dotted lines in the figure. The first one is *R*_1_ = 175 nm and *H*_1_ = 275 nm and corresponds to the magnetic dipole resonance. The second is *R*_2_ = 260 nm and *H*_2_ = 205 nm and corresponds to the overlap between an electric anapole state and a magnetic dipole. These two sizes show a high level of absorption for the metasurface. An absorption enhancement can improve the quantum efficiency and optical responsivity of the device. Therefore, we considered two metasurfaces: one with nanodisks of *R*_1_ = 175 nm and *H*_1_ = 275 nm and the second with nanodisks of *R*_2_ = 260 nm and *H*_2_ = 205 nm and studied their optical and electrical characteristics.

To gain insight into how the dimensions of the device affect its light absorption, the scattering of the light wave by an individual Ge nanodisk was studied by employing the Cartesian multipole expansions [31,32]. In this analysis, the incident light induces current densities inside the nanodisk that, in turn, produces the electromagnetic field. Unlike the spherical multipoles representation, Cartesian decomposition explicitly includes the toroidal multipole’s contributions in addition to the electric and magnetic contributions. The scattering cross-section $\sigma_{sca}$, normalized to the incident energy flux ${\left(\varepsilon_{0}\varepsilon_{d}/\mu_{0}\right)}^{1/2}{\left|E_{inc}\right|}^{2}/2$, as a combination of different multipoles is expressed as [33,34]

(1) $$\sigma_{sca}\approx \frac{k_{0}^{4}}{6\pi \varepsilon_{0}^{2}{\left|E_{inc}\right|}^{2}}{\left|p+\frac{ik_{d}}{v_{d}}T\right|}^{2}+\frac{k_{0}^{4}\varepsilon_{d}\mu_{0}}{6\pi \varepsilon_{0}{\left|E_{inc}\right|}^{2}}{\left|m\right|}^{2}\;+\frac{k_{0}^{6}\varepsilon_{d}}{720\pi \varepsilon_{0}^{2}{\left|E_{inc}\right|}^{2}}\sum_{\propto \beta }{\left|Q_{\propto \beta }\right|}^{2}+\frac{k_{0}^{6}\varepsilon_{d}^{2}\mu_{0}}{80\pi \varepsilon_{0}{\left|E_{inc}\right|}^{2}}\sum_{\propto \beta }{\left|M_{\propto \beta }\right|}^{2},$$

where $k_{0}$, $\varepsilon_{0}$, and $\mu_{0}$ respectively refer to angular wavenumber, electric permittivity, and magnetic permeability in free space, while $\varepsilon_{d}$ refers to the relative permittivity of the surrounding medium. The first term includes moments of electric dipole ED and toroidal dipole TD (i.e., p and T, respectively) and their interference ($Im\left(p^{†}.T\right)$). The destructive interference of far-field contributions of these electric and toroidal dipoles results in a cancellation of the scattering, while the near-field is still non-zero. Such configurations are known as anapole states [10]. On the other hand, the interference can be constructive, forming a super dipole [35]. The moments of a magnetic dipole (MD), electric quadrupole (EQ), and magnetic quadrupole (MQ) are also shown by $m,\;Q_{\propto \beta },M_{\propto \beta }$, respectively.

**Figure 3:**
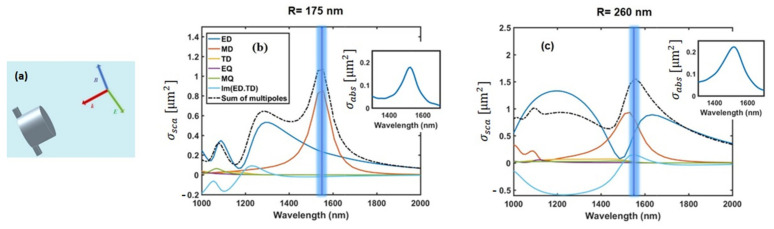
(**a**) Schematic of the plane wave light illuminating a single Ge nanoparticle. (**b**,**c**) excited modes in the single Ge nanoparticle when *R* = 175 (*H* = 275 nm) and when *R* = 260 nm (*H* = 205 nm). The vertical blue lines point to the wavelengths around 1550 nm (C-telecommunication band).

The excited modes contributing to the scattering cross-section of a single Ge nanoparticle are depicted in Figure 3a–c. As seen from Figure 3b,c, the TD and its interference with the ED become important for the blue side of the spectrum, particularly for wavelengths shorter than 1550 nm. Our multipolar analysis also shows a separation of ED and MD resonance peaks with the decrease of the disk radius *R*. As can be noticed from the insets of Figure 3b,c, the resonant peaks in the absorption cross-section ($\sigma_{abs}$) spectra are observed in the wavelength region of 1500–1550 nm. The resonant peak for the case of *R* = 175 nm (*H* = 275 nm) is mainly referred to as the MD contribution, as it has been shown that the MD excitation leads to the enhancement of the light absorption via the localization of electromagnetic energy inside dielectric nanoparticles [36,37]. For the particle with *R* = 260 nm (*H* = 205 nm), the resonant peak comes from the coupling between the magnetic dipole and the electric anapole state.

**Figure 4:**
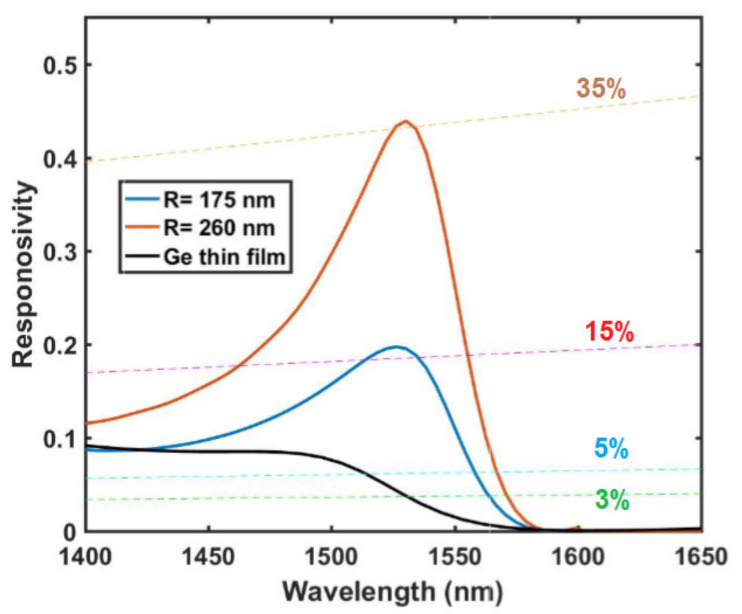
Normalized responsivity as a function of the incident light wavelength for the two devices of *R* = 175 nm (*H* = 275 nm) and *R* = 260 nm (*H* = 205 nm) and for 275 nm-thick Ge film. Dashed lines show the various quantum efficiencies across the whole wavelength range (i.e., Responsivity = η qλ/hc where η = 0.03, 0.05, 0.15, and 0.35).

To calculate the quantum efficiency of the optimized device, simulations were performed using Lumerical’s FDTD Simulation Suite for a single unit-cell with periodic boundary conditions in the *x-y* plane and perfectly matching layer (PML) for the *z*-direction. The optical responsivity of the device, then, was calculated as a function of the incident photon wavelength λ, by the relation [38].

(2) $$Responsivity\;=\;\eta \frac{q\lambda }{hc}$$

where q and h, respectively refer to the elementary charge and Plank constant, c indicates the speed of light in a vacuum, and the quantum efficiency, η, is equal to the normalized absorption in the Ge region with the assumption that all absorbed light in the depletion layer of the PIN diode converts into collectible hole-electron pairs (internal quantum efficiency of 100%). We calculated the responsivity of our metasurface for two different sizes of the disk: *R* = 175 nm (*H* = 275 nm) and *R* = 260 nm (*H* = 205 nm). Both designs have a lattice constant of *a* = 900 nm. As shown in Figure 4, the device shows resonant quantum efficiencies of 15% to 35% at the C-band.

It is also useful to compare these quantum efficiencies with that of an unstructured Ge layer. As can be seen from Figure 4, a uniform 275 nm-thick Ge film has a quantum efficiency of under 3% at this wavelength range. In contrast, our resonant dielectric design significantly enhances the light absorption due to the superposition of the magnetic dipole and the electric anapole excitation.

### 3.2. Electrical Response

**Figure 5:**
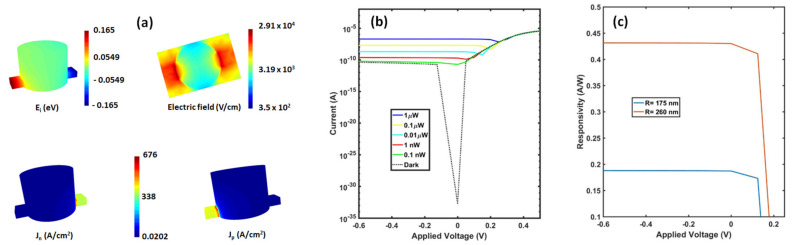
Electrical response of the photodetector. Panel (**a**) shows intrinsic energy level E_i_, electric field magnitude at xy-plane, and electron (hole) current density Jn (Jp) at zero bias for the device of *R* = 175 nm (*H* = 275 nm). (**b**) Current-voltage characteristics for the device of *R* = 175 nm (*H* = 275 nm) at dark and at various illumination. (**c**) responsivity of the device of *R* = 175 nm (*H* = 275 nm) and of *R* = 260 nm (*H* = 205 nm).

Electrical analysis of a single unit cell of the device was conducted by the finite element method (FEM) using Lumerical’s DEVICE Simulation Suite to calculate the current-voltage characteristic (I-V curve) and bandwidth at room temperature, 300 K. Some electronic properties of the device of *R* = 175 nm (*H* = 275 nm) including intrinsic energy level E_i_ (i.e., the Fermi energy of an undoped semiconductor), electric field magnitude at the xy-plane, and electron (hole) current density Jn (Jp) at zero bias have been illustrated in panel (a) of Figure 5. The I-V curves of the device *R* = 175 nm (*H* = 275 nm) in the dark and under illumination are illustrated in Figure 5a. The I-V curve was calculated for a range of optical powers (0.1 nW−1 μW) reaching the single unit cell of the device. We used small power to ensure that the photocurrent linearly increases with the incident power, and thus the photodetector does not suffer from the saturation optical power resulting from physical mechanisms including thermal and space-charge effect [39,40]. On the assumption that the detector can ideally suppress majority charge carriers to an undetectable level, the dark current comes from the surface states and the bulk minority carrier diffusion including Shockley–Read–Hall, radiative, and Auger generation [41]. As seen from Figure 5, the dark current is in order of $\sim 10^{-11}\;A$ for the single unit cell. However, this value can in reality become larger due to the imperfections present in real semiconductor structures. As expected, the dark current at zero voltage bias approaches zero because the diode equation $I\left(V\right)\;\sim \;\left(e^{\mathrm{eV}/\mathrm{kT}}-1\right)$ [42] predicts there is no current at zero voltage. For an optical power of 0.1 nW, the current in the device is comparable to the dark current, while it goes up to ~0.1 μA when power increases to 1 μW. We also illustrated the responsivity (photocurrent/incident power) for the devices with *R* = 175 nm (*H* = 275 nm) and *R* = 260 nm (*H* = 205 nm) in Figure 5b being respectively 0.19 and 0.43 A/W which is comparable to that of the typical normal incidence photodetectors (see Table 1). The responsivity at zero bias (0 V) is ∼99% of that at −0.5 V. This means that most photogenerated charge carriers are collected without applying any bias. The development of PIN photodetectors working at zero bias is of interest [43] because the removal of the DC bias circuit from the detectors decreases cost, dimensions, and even the sophistication of the circuitry. It also eliminates the noise of leakage current coming from a finite bias.

Photodetectors can be used to measure the time and frequency response of an optical system, for instance, the data stream of a communication system. To increase the operating speed of optical signal processing, high-speed photodetectors are needed. With the development of short-pulse lasers, optical signals can be significantly faster. Still, a high-speed photodetector for signal processing is also required to perform a fast conversion of an optical to an electrical signal. Two factors limiting the bandwidth are: (i) the transit time of drift carriers ($T_{tr}\;=\;v_{s}/L$), that is the time taken by generated charge carriers to travel across the depletion zone and get swept in electrical contacts, and (ii) the RC filter circuit with a 3 dB cutoff frequency of $f_{RC}\;=\;1/2\pi rC$ where C and r are the capacitance and resistance of the device.

The bandwidth corresponding to the RC component is obtained via the capacitance and resistance of the device calculated by small-signal analysis. To calculate the capacitance of the collection layer, the device was considered at a reverse bias of 0.6 V and a small-signal voltage $v_{ac}\;=\;0.001\;V$ was applied. The small-signal current $i_{ac}$ was then calculated by the finite element method to obtain the admittance $y\;=\;i_{ac}/v_{ac}$. The capacitance was finally obtained by $C\;=\;Im\left(y\right)/\omega$ as a function of the frequency ω. It is worth mentioning that the capacitance of a PIN diode is not dependent on the applied bias due to the fixed size of the intrinsic layers. The capacitance is significantly smaller ($\sim 10^{-18}\;F$) over the range of frequency, therefore despite the large series resistance ($r\;\sim \;10^{5}\;\Omega$) the bandwidth of the device is limited by the transit-time bandwidth, $f_{3dB}$. Given $f_{3dB}≅0.382v_{s}/L$, the maximum values of $f_{3dB}$ for devices of *R* = 175 nm (*H* = 275 nm) and *R* = 260 nm (*H* = 205 nm) are predicted to be 65 and 44 GHz, respectively. To simulate the bandwidth of the photodetector, the impulse response that describes the response of the detector to an input pulse as a function of time was calculated. The frequency response of the photodetector was then calculated by a Fourier transform of the impulse response to obtain $f_{3dB}$ under different applied voltages.

**Figure 6:**
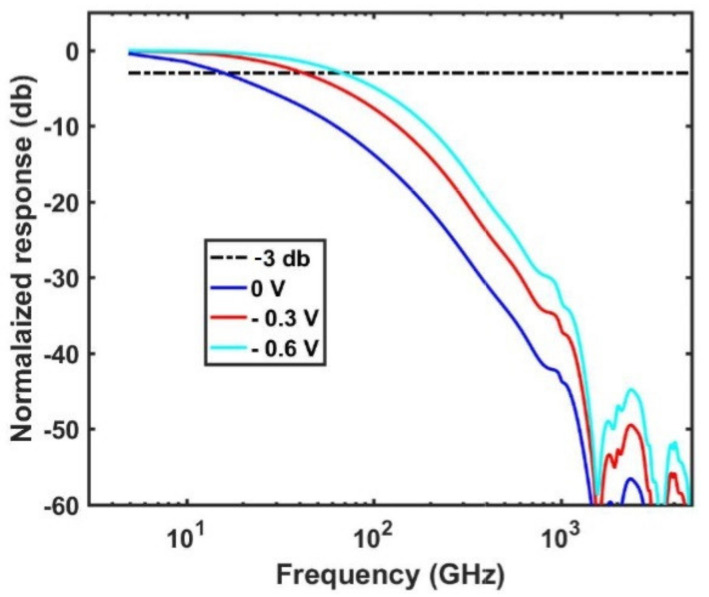
The transit-time bandwidths of the device (*R* = 175 nm and *H* = 275 nm) at 0 V, −0.3 V, and −0.6 V are 16, 42, and 65 GHz, respectively.

The $f_{3dB}$ for *R* = 175 nm (*H* = 275 nm) was calculated for the various applied biases and is presented in Figure 6. The figure shows that a high bandwidth of 65 GHz is obtained at −0.6 V. Typically for low power consumption, the reverse bias of the photodetectors should be less than −1.5 V. The bandwidth at zero bias is 16 GHz which indicates the possibility of zero-bias operation. For the device of *R* = 260 nm (*H* = 205 nm), a bandwidth of 44 GHz is obtained at −0.5 V.

We showed that the dielectric metasurfaces may enable us to design high-speed normal incidence photodetectors only by nanostructuring their active region. At the same time, the responsivity remains at a level comparable to the traditional normal incidence photodetectors despite the subwavelength dimensions of the region. Our metasurface also permits tuning the excited optical modes and their spectral overlap to achieve tunable resonances in the responsivity of the device and allows for spectrally selective light detection. Therefore, it could be applied for narrowband photodetection [16] and filter-free narrowband light detection [44] which are crucial for NIR imaging and machine vision [45]. In this study, we have shown the possibility of a bandwidth of 44–65 GHz and responsivity between 0.19 and 0.43 A/W. Still, our results suggest that a higher bandwidth and responsivity could be achieved by changing the geometry and arrangement of the Ge nanoparticles (e.g., a metasurface made of nanocubes or a metasurface in which each unit cell consists of two nanodisks). It may also promise the extension of Ge wavelength cut-off beyond 1550 nm which would enable Ge photodetectors to be comparable to their counterparts that are based on InGaAs alloys. Due to the subwavelength size of the active area, the detectors can exhibit a minimal junction capacitance; thus, the bandwidth of the detector is not limited by RC time. On the other hand, such a small size leads to high shunt resistance which promises devices with lower thermal noise.

## 4. Conclusions

A lateral PIN Ge photodetector was investigated for the realization of fast IR photodetection based on efficient light absorption in nanostructured semiconductors. Each PIN pixel is made of a Ge nanodisk sandwiched between two n- and p-doped Ge nanostructures while metal contact circuits are embedded in SiO_2_ substrate. Efficient light absorption inside the Ge nanodisks that results from the efficient coupling of light to the Ge nanodisks is demonstrated through the engineering of the nanodisk’s resonant optical modes. This efficient coupling comes from the resonant magnetic response of the Ge nanoparticle that has a comparable strength with its resonant electric response. The photodetector exhibits small junction capacitance and high shunt resistance, promising a device with low thermal noise. The device exhibits a high bandwidth of 65 GHz at the reverse bias of 0.6 V. It also shows a zero-bias operation with a responsivity that is over 99% of that at −0.5 V.

## Data Availability

Data is contained within the article.
